# Glutaminase inhibition in renal cell carcinoma therapy

**DOI:** 10.20517/cdr.2018.004

**Published:** 2019-06-19

**Authors:** Aleksandra M. Raczka, Paul A. Reynolds

**Affiliations:** ^1^School of Medicine, University of St Andrews, St Andrews KY16 9TF, UK.; ^2^Biomedical Sciences Research Complex, University of St Andrews, St Andrews, UK.

**Keywords:** Glutamine addiction, renal cell carcinoma, combination therapy, metabolism

## Abstract

Receptor tyrosine kinase inhibitors have been a standard first-line therapy for renal cell carcinoma (RCC) for over a decade. Although they stabilize the disease, they are unable to remove all tumor cells, leading to relapse. Moreover, both intrinsic and acquired resistance to therapy are a significant health burden. In order to overcome resistance, several combination therapies have been recently approved by the FDA. Another approach takes advantage of altered metabolism in tumor cells, which switch to alternative metabolic pathways to sustain their rapid growth and proliferation. CB-839 is a small molecule inhibitor of kidney type glutaminase (GLS). GLS is often upregulated in glutamine addicted cancers, enhancing glutamine metabolism for the production of energy and the biosynthesis of various cellular building blocks. CB-839 is currently in clinical trials for several tumors, including clear cell (cc)RCC, both as monotherapy and in combination with the approved therapeutic agents everolimus, cabozantinib and nivolumab. Early results of Phase 1/2 clinical trials look promising, especially for CB-839 plus cabozantinib, and all combinations seem to be well tolerated. However, cancer cells can activate compensatory pathways to overcome glutaminolysis inhibition. Therefore, genetic and metabolomic studies are crucial for the successful implementation of CB-839 alone or in combination in subgroups of ccRCC patients.

## Introduction

Renal cell carcinoma (RCC) represents 90% of kidney cancers and 2%-3% of all malignancies. It remains the deadliest genitourinary malignancy, with a new case count globally of approximately 270,000 and over 100,000 deaths annually^[1-3]^. Surgery can be performed for early stage localized tumors, however 30% of patients present with metastases at the time of diagnosis^[4]^. Of those who present with non-metastatic RCC, many will develop tumors at distant sites, such as brain, lungs, lymph nodes, liver and bone. At this stage, the 5-year survival rate is 0%-20%^[5]^. Drug resistance to currently available therapies is a significant issue in the treatment of RCC and can be attributed to very high intra-tumoral heterogeneity (ITH)^[6]^. It is estimated that 25%-30% of patients presenting with advanced RCC do not respond to antiangiogenic drug therapy from the beginning, which results in a poor prognosis. Of those who initially respond, most develop resistance within 6-15 months after the start of treatment^[7-9]^. Current efforts aiming at overcoming these issues involve combination therapies with already approved targeted therapies and checkpoint inhibitors, as well as therapies targeting altered tumor metabolism.

## Current state of available therapies

### Monotherapies

From the early 1990s until 2006, RCC was treated with interferon-α or interleukin-2 (IL-2). These treatments had low rates of efficacy (5%-20%), high morbidity and mortality and are rarely used today. A multi-target receptor tyrosine kinase (RTK) inhibitor, sunitinib, was approved by the FDA in 2006 and started the era of targeted therapy for RCC^[10,11]^. Until 2009, when the FDA approved everolimus as a second-line therapy, no drugs were available for patients whose disease progressed after initial treatment^[12,13]^. Other approved RTK inhibitors include bevacizumab, axitinib and lenvatinib, all inhibiting vascular endothelial growth factor receptors (VEGFR). Pazopanib is another approved small molecule, which works as a multityrosine kinase inhibitor. Additionally, cabozantinib inhibits AXL, RET, MET, KIT, FLT3, ROS1, TYRO3 and TRKB, as well as VEGFR^[12]^. Mammalian target of rapamycin (mTOR) is a serine/threonine-specific protein kinase frequently mutated in RCC. Examples of mTOR inhibitors include everolimus and temsirolimus^[14]^. Ipilimumab is designed to target Cytotoxic T-Lymphocyte Associated Protein 4 (CTLA-4), a protein receptor which downregulates the immune system by inhibiting T cell proliferation and IL-2 production. Inhibition of CTLA-4 leads to reprogramming of T cell metabolism, thus activating these cells. Several programmed death-1 (PD-1) receptor inhibitors, such as nivolumab, pembrolizumab and ateozumab, work to increase T cell metabolism^[12,15]^. Despite significant progress in the field, drug resistance remains a significant health burden^[12]^.

### Combination therapies

Combination therapy is a treatment approach where two or more therapeutics are used together to treat a disease. It was first applied to treat cancer in 1965, when Emil Frei, James F. Holland and Emil J. Freirich noticed that the treatment of acute leukemia in children using methotrexate, 6-mercaptopurine plus vincristine had an additive effect on disease remission^[16]^. Since then, combination therapies with drugs that target different biochemical pathways have been of great focus in cancer research. The objective of these studies is to find combinations which have synergistic or additive effects. More recently, efforts have been made to develop restrictive combinations, which spare healthy cells but specifically target tumor cells. This approach takes advantage of differences between healthy and malignant cells, such as the absence of a drug target. This is exemplified by the absence of functional p53 in some tumor cells, where growth of normal cells is arrested by one drug in order to protect them from the effect of another drug, which only targets actively dividing cells^[17]^. Drug repurposing is another approach where drugs already approved for use in treatment of different diseases could be employed in the cancer setting^[18]^.

Development of combination therapies is on the increase and could one day become a new standard of care. Its superiority over monotherapy presents in a potential decrease in drug resistance with concurrent therapeutic benefit, such as reduced tumor growth and prevention of metastases. The first FDA-approved combination for advanced RCC was lenvatinib plus everolimus as second-line therapy. The approval was based on a better outcome over therapy with a single agent and acceptable toxicity^[19]^. Similarly, following Phase II clinical trials, bevacizumab plus INFα is one of the available first-line treatments in patients presenting with metastatic disease^[20,21]^. Published in April 2018, the Phase III CheckMate 214 study of nivolumab plus ipilimumab *vs.* sunitinib led to the FDA approval of the combination therapy for treatment-naive intermediate- and poor-risk patients with advanced RCC. In this study, the combination produced a more favorable overall survival (OS) and a higher objective response rate (ORR) in the above-mentioned patient groups, but not in the low-risk group. Likewise, it improved progression-free survival (PFS) in comparison to sunitinib, however these results did not meet the prespecified statistical significance (*P* = 0.009)^[22,23]^.

One of the treatments awaiting approval by The National Institute for Health and Care Excellence (NICE) is atezolizumab plus bevacizumab, which was shown to improve PFS as a first-line treatment of advanced or metastatic RCC in comparison to sunitinib^[24]^. A Phase Ib/II study of lenvatinib plus pembrolizumab involved 30 patients with metastatic RCC, of which 21 (70%) developed grade 3 or 4 adverse events (AEs) and four discontinued treatment due to the AEs^[24]^. This study reported manageable AEs and did not identify any new safety concerns. A Phase III study of this combination *vs.* sunitinib is underway^[25]^. Combinations of cabozantinib (Cabo) plus nivolumab (Nivo) and CaboNivo plus ipilimumab were tested in a Phase I study and its expansion trial. Both combinations were evaluated for favorable efficacy and tolerability in patients with mRCC and other genitourinary malignancies^[26,27]^. Safety and toxicity of tivozanib plus nivolumab were ruled to be acceptable in a Phase Ib study^[28]^. Of 18 patients, all developed at least one AE and five (38%) showed signs of grade 3-4 AE. Additional patients are currently being enrolled and treated in a Phase II trial that aims to evaluate combination efficacy^[28]^.

## Novel approaches for the treatment of RCC

### Glutamine addiction

Over the past few years, cancer cell metabolism has gained significant interest in the research community and metabolic reprogramming is now recognized as one of the hallmarks of cancer^[29]^. In order to sustain their rapid growth, malignant cells switch to alternative metabolic pathways, which allow them to increase energy and nutrient production. Cancer cells are largely dependent on aerobic glycolysis, also known as “the Warburg effect”, whereas normal cells generate energy by oxidative phosphorylation in mitochondria. Malignant cells may also rely on a non-essential amino acid, glutamine, which can be as equally important as glucose for the survival of tumor cells^[30]^. In the kidney, glutamine is converted into glutamate by the mitochondrial enzyme glutaminase (GLS1). Glutamate can then enter Krebs cycle and act as an intermediate for biosynthesis of various molecules, such as nucleotides, amino acids and glutathione^[31]^. Due to its involvement in the pathways mentioned above, glutamine plays an important role in cell proliferation and protection against oxidative stress^[30]^. Moreover, depleted glutamine levels are often found in the tumor environment caused by its uptake into cancer cells. This in turn affects T-cell growth, proliferation and cytokine production, all of which require high energy input. Glutamine metabolism is thought to be of particular importance in T-cell activation^[31]^.

The first small molecule GLS inhibitors, such as 6-diazo-5-oxo-l-norleucine (DON), were first described in the 1950s and suggested to be useful in chemotherapy. Such early compounds also included azaserine and acivicin^[32,33]^. All three drugs are glutamine analogues and bind irreversibly to the active site of the enzyme, thus inhibiting glutamine binding. Despite promising *in vitro* data, clinical studies with these compounds have been restricted due to limited therapeutic activity and severe toxicities^[33,34]^. Dose-limiting adverse events are likely due to low selectivity and inhibition of other glutamine-utilizing enzymes, such as amidotransferases and glutamine synthetase. However, DON has recently been used in clinical studies in patients with advanced refractory solid tumors, where its combination with PEGylated glutaminase showed therapeutic activity and a tolerable toxicity profile^[35]^.

Bis-2-(5-phenylacetamido-1,2,4-thiadiazol-2-yl)ethyl sulfide 3 (BPTES) is an example of the class of allosteric inhibitors of GLS1. It binds to both free enzyme and enzyme-substrate complexes and forms a stable but inactive tetramer complex^[36]^. In addition to its high specificity, it is also more effective in inhibiting GLS1 that DON, with a Ki of 3 µmol/L^[37]^. Therefore, it is less likely to interact with transporters, receptors or other molecules that recognize glutamine or glutamate as their substrates. While it was shown to slow cancer cell proliferation *in vitro* and in xenografts^[38]^, its moderate potency (IC_50_ = 3.3 μmol/L), poor metabolic stability and low solubility, limit its potential use in the clinical setting^[39]^.

### CB-839 *in vitro*

**Figure 1:**
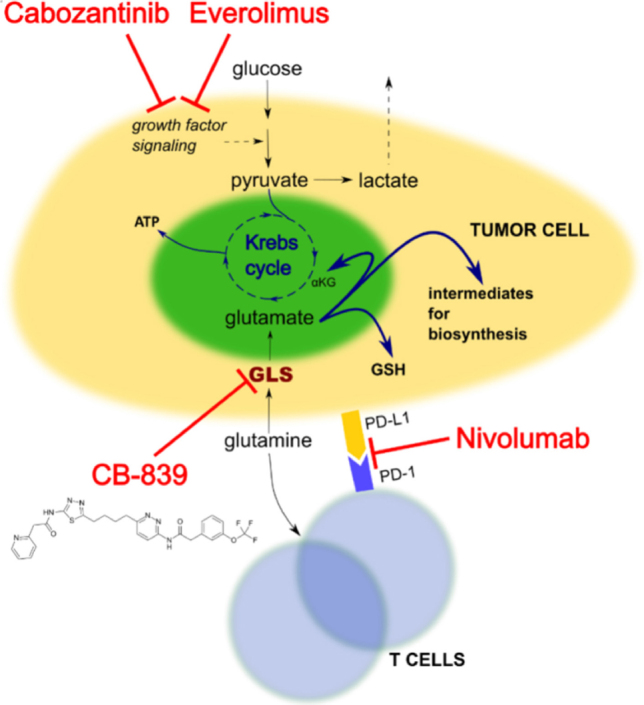
Pathways inhibited by CB-839, nivolumab, cabozantinib and everolimus. GLS: glutaminase; GSH: glutathione; ATP: adenosine triphosphate; αKG: alpha-ketoglutarate; PD-1: programmed death receptor 1; PD-L1: programmed death ligand 1

CB-839 is a small, orally administered molecule, which inhibits human glutaminase in an allosteric and reversible manner [Figure 1]. It exerts a dual action by inhibiting tumor cells and activating T cells^[40]^. It was shown to downregulate mTOR signaling in RCC cell lines and RMPI8226 myeloma cells, as shown by a decrease in proteins such as phospho-mTOR and phospho-S6. The same study found a reduction in oncogenic proteins c-Myc and c-Kit and an increase in programmed cell death proteins, such as cleaved-PARP^[41]^. It also inhibits glutaminase in triple negative breast cancer (TNBC) cell lines, which are highly sensitive to glutaminolysis inhibition^[42,43]^. CB-839 exhibits high specificity towards GLS1, demonstrated by suppression of ATP production, biosynthesis and maintenance of redox balance, in which glutamine acts as an intermediate^[42]^. CB-839 also has antitumor activity *in vivo*, as shown by two studies of xenograft models of TNBC^[42]^. In a patient-derived xenograft model, administration of 200mg/kg of CB-839 twice a day resulted in 61% tumor growth suppression when compared to untreated control. The HER2 + basal-like cell line JIMT-1 was used in a cell-line based xenograft model. Two treatment regimens were evaluated: CB-839 on its own or in combination with the TNBC standard therapy paclitaxel. Administration of paclitaxel alone led to tumor reduction followed by tumor expansion, whereas CB-839 alone or in combination with paclitaxel reduced tumor growth with no tumor regrowth over time^[42]^.

The activity of CB-839 was tested in a range of kidney cancer cell lines. Exposure to 1 µmol/L CB-839 for 72h resulted in varying degrees of death of most but not all ccRCC lines tested, including 786-O, 769-P and A498. This variation was not associated with VHL mutant or wild-type status. In contrast, CB-839 did not seem to affect growth of six non-RCC cell lines, such as G402 and JMU-RTK2^[44]^. Other studies propose that glutamine becomes the preferred substrate for lipogenesis in VHL mutant ccRCC lines^[45]^, suggesting that glutaminase inhibition should be cytotoxic in these cells.

### CB-839 monotherapy

CB-839 safety and tolerability in solid tumors is currently being evaluated in a first-in-man study estimated to be completed in September 2019 (ClinicalTrials.gov Identifier: NCT02071862)^[46]^. The study design contains both monotherapy and combination arms. As part of the combination, patients will receive CB-839 with drugs currently used for treatment of RCC, TNBC, NSCLC, mesothelioma and tumors with mutations in enzymes of the Krebs cycle. This multicenter, open-label, dose escalation study includes patients presenting with metastatic or locally advanced tumors. As monotherapy, patients will receive 100-800 mg CB-839 twice- or three times daily in cycles lasting for 21 days. The 600 mg twice-daily was selected as the dose administered to the expansion cohort. In 2016, Calithera reported that 4 out of 75 patients experienced grade 3 or 4 AEs. Out of 19 patients with several previous therapies (median = 3 therapies), 10 were ccRCC patients of which one achieved a confirmed ongoing partial response as of 8.3 months of therapy. To date, the study showed that continuous administration of CB-839 is characterized by an acceptable safety profile. Moreover, it significantly inhibits glutaminase and shows promising signs of clinical activity in various tumors, including RCC^[47,48]^.

### CB-839 plus everolimus

Everolimus is an inhibitor that targets the kinase mTOR, which is a part of the PI3K/AKT/mTOR pathway [Figure 1], which is frequently mutated in RCC. Its crosstalk with VHL/HIF creates a positive feedback loop, where HIF-induced expression of growth factors activates PI3K/AKT pathway through RTKs. This in turn activates mTORC1 and mTORC2 leading to upregulation of HIF^[49]^. HIF translation and the consequent angiogenesis can be inhibited by repression of mTOR^[50]^.

*In vitro* studies were performed using the ACHN cell line, which is thought to represent papillary RCC given its chromosomal aberration profile, characteristic c-met polymorphism and WT VHL status^[51,52]^. Five drug concentration combinations were evaluated: CB-839 at 18.8-300 nmol/L with everolimus at 1.6-100 nmol/L. For all combinations, cell survival was lower when compared to monotherapy. Pre-clinical studies of Caki-1 xenografts showed that monotherapy with CB-839 or everolimus, resulted in tumor growth inhibition (TGI) of 51% and 52%, respectively. However, the combination of the two gave TGI of 85%^[48]^.

An ongoing clinical study (ClinicalTrials.gov Identifier: NCT02071862) builds on these pre-clinical data, where the two drugs synergize and inhibit glutamine and glucose metabolism when combined^[46]^. So far it has shown that the combination is well tolerated in ccRCC and papillary carcinoma patients and CB-839 does not increase the toxicities, compared to everolimus alone. The most common AEs were nausea and fatigue. A single case of dose-limiting toxicity was attributed to everolimus and after lowering the dose, the patient remained on study^[48]^. A 100% disease control rate (DCR) was reported for ccRCC patients (12/12) and 67% for papillary RCC patients (2/3). Preliminary PFS for both RCC histotypes was 8.5 months^[48,53]^.

### CB-839 plus nivolumab

PD-1 is an inhibitory receptor expressed on activated T cells, which normally functions to dampen the immune response. PD-1 is engaged by PD-L1 (and PD-L2) expressed by tumor cells and infiltrating immune cells. Inhibition of the interaction between PD-1 and PD-L1 enhances anti-tumor responses, delays tumor growth, and may facilitate tumor rejection. Nivolumab is a fully humanized IgG4 PD-1 immune checkpoint inhibitor, which prevents ligand binding to PD-1 and promotes antitumor immunity by T cells [Figure 1]^[54]^. T cells require glutamine for proliferation and compete with glutamine-addicted tumors for this amino acid, creating a metabolic checkpoint^[31]^. In contrast to reduced glutamine availability, GLS inhibition by CB-839 does not affect T cell activation, their entry into S phase or their division *in vitro*. Instead, CB-839 reduces the use of glutamine by malignant cells relieving the metabolic checkpoint and making glutamine available for T cells. CB-839 is proposed to synergize with nivolumab in syngeneic colon tumor mouse models to improve T cell activation and proliferation, boosting their anti-cancer activity^[55]^.

A Phase 1/2 study of CB-839 plus standard of care nivolumab is currently open and recruiting patients (ClinicalTrials.gov Identifier: NCT02771626). It aims to evaluate safety, pharmacokinetics and pharmacodynamics of the combination in patients with ccRCC, melanoma and NSCLC. Patients are receiving escalating doses of CB-839 twice daily and standard dose nivolumab to determine recommended Phase 2 dose (RP2D). In Phase 2, patients are separated into five cohorts: ccRCC naïve to checkpoint inhibitors; ccRCC recently treated with nivolumab; ccRCC, melanoma or NSCLC with prior PD-1 therapy. Study completion is expected in June 2019^[56]^.

### CB-839 plus cabozantinib

Cabozantinib is a signal transduction inhibitor and its targets include VEGFR, ARK and MET [Figure 1]. Early pre-clinical studies using the Caki-1 cell line and Caki-1 xenografts showed superiority of cabozantinib plus CB-839 over monotherapy when measuring its anti-proliferative effect. The combination reduced signal transduction more than cabozantinib alone by reducing pERK and pAKT proteins^[57]^. The two drugs also synergize to inhibit TCA cycle activity as measured by oxygen consumption rate. In xenograft models, where tumors of approximately 400 mm^3^ were treated with DMSO, CB-839, cabozantinib or a combination of the drugs, monotherapy resulted in slower tumor growth, which was enhanced in combination models^[57]^.

Following *in vitro* and mouse studies, 13 patients with ccRCC or papillary RCC were enrolled into a Phase 1 clinical trial. RP2D was evaluated at 800mg and no maximum tolerated dose was reached. Study outcomes including ORR of 40% and DCR of 100% in ccRCC, favorable safety profile and zero patients with PD, led to a randomized Phase 2 study, CANTANA^[58]^. This study is currently recruiting patients and aims to compare cabozantinib plus CB-839 *vs.* cabozantinib plus placebo in 298 patients with previous 2 or 3 lines of therapy. The key endpoints are PFS and OS with completion expected in 2022^[58]^.

## Conclusions and future directions

The development of targeted therapy for RCC has revolutionized its treatment. RCC tumors are highly vascularized and current standard of treatment focuses mainly on drugs targeting angiogenesis by inhibiting VEGF and mTOR. However, high ITH and intrinsic and acquired therapy resistance prevents removal of all malignant cells. Residual tumor cells expand and lead to clinical relapse, creating the need for second- and further line therapies. Several combination therapies have been recently approved, however they are still in their early stages of use. Moreover, checkpoint inhibitors have not been used in the clinical setting to treat mRCC for long enough to fully evaluate their efficacy. Many RCC tumors are now being recognized to have altered metabolism in comparison to normal cells. Although efforts to formulate efficient glutaminase inhibitors are not a completely new concept, they are yet to be approved for use in the clinical setting. CB-839 has been shown to be well tolerated, but as monotherapy, its preliminary efficacy is limited to achieving long-term stable disease. This suggests that it may be most effective in combination with other agents. In April 2018, the FDA granted Fast Track designation for CB-839 plus Cabo based on encouraging activity from a Phase 1 trial in heavily pre-treated mRCC patients. Combination therapy of CB-839 with everolimus requires more data to assess its efficacy. Since there is such a significant burden of resistance to currently available treatments, resistance may also be an issue for new treatments. Indeed, metabolomic studies of an allosteric and reversible inhibitor of GLS, C.968, found that lipid catabolism is activated to compensate for inhibition of glutaminolysis in several cancer cells^[59]^. This suggests that further genetic and metabolomic studies will be crucial for the successful implementation of CB-839 into routine clinical practice.

## Declarations

### Copyright

© The Author(s) 2019.
